# Glycogen Metabolism Impairment via Single Gene Mutation in the *glgBXCAP* Operon Alters the Survival Rate of *Escherichia coli* Under Various Environmental Stresses

**DOI:** 10.3389/fmicb.2020.588099

**Published:** 2020-09-25

**Authors:** Mengmeng Wang, Qinghua Liu, Xingxing Kang, Zuobin Zhu, Huan Yang, Xiangyu Xi, Xiao Zhang, Yan Du, Mengzhe Guo, Daoquan Tang, Liang Wang

**Affiliations:** ^1^Jiangsu Key Laboratory of New Drug Research and Clinical Pharmacy, School of Pharmacy, Xuzhou Medical University, Xuzhou, China; ^2^Department of Pharmaceutical Analysis, School of Pharmacy, Xuzhou Medical University, Xuzhou, China; ^3^Department of Bioinformatics, School of Medical Informatics and Engineering, Xuzhou Medical University, Xuzhou, China; ^4^Department of Genetics, School of Life Sciences, Xuzhou Medical University, Xuzhou, China; ^5^School of Laboratory Medicine, Xuzhou Medical University, Xuzhou, China; ^6^Xuzhou Infectious Disease Hospital, Xuzhou, China

**Keywords:** glycogen, bacterial viability, environmental endurance, biofilm, low temperature

## Abstract

Glycogen is a highly branched polysaccharide that is widely present in all life domains. It has been identified in many bacterial species and functions as an important energy storage compound. In addition, it plays important roles in bacterial transmission, pathogenicity, and environmental viability. There are five essential enzymes (coding genes) directly involved in bacterial glycogen metabolism, which forms a single operon *glgBXCAP* with a suboperonic promoter in *glgC* gene in *Escherichia coli*. Currently, there is no comparative study of how the disruptions of the five glycogen metabolism genes influence bacterial phenotypes, such as growth rate, biofilm formation, and environmental survival, etc. In this study, we systematically and comparatively studied five *E. coli* single-gene mutants (Δ*glgC*, Δ*glgA*, Δ*glgB*, Δ*glgP*, Δ*glgX*) in terms of glycogen metabolism and explored their phenotype changes with a focus on environmental stress endurance, such as nutrient deprivation, low temperature, desiccation, and oxidation, etc. Biofilm formation in wild-type and mutant strains was also compared. *E. coli* wild-type stores the highest glycogen content after around 20-h culture while disruption of degradation genes (*glgP*, *glgX*) leads to continuous accumulation of glycogen. However, glycogen primary structure was abnormally changed in Δ*glgP* and Δ*glgX*. Meanwhile, increased accumulation of glycogen facilitates the growth of *E. coli* mutants but reduces glucose consumption in liquid culture and *vice versa*. Glycogen metabolism disruption also significantly and consistently increases biofilm formation in all the mutants. As for environmental stress endurance, glycogen over-accumulating mutants have enhanced starvation viability and reduced desiccation viability while all mutants showed decreased survival rate at low temperature. No consistent results were found for oxidative stress resistance in terms of glycogen metabolism disruptions, though Δ*glgA* shows highest resistance toward oxidation with unknown mechanisms. In sum, single gene disruptions in *glgBXCAP* operon significantly influence bacterial growth and glucose consumption during culture. Accumulation and structure of intracellular glycogen were also significantly altered. In addition, we observed significant changes in *E. coli* environmental viabilities due to the deletions of certain genes in the operon. Further investigations shall be focused on the molecular mechanisms behind these phenotype changes.

## Introduction

Glycogen is a homogeneous polysaccharide with a typical feature of a highly and randomly branched structure, which is widely spread across the domains of life (Wang and Wise, 2011; Wang et al., 2020). It consists of glucosyl residues that are linked together by α-1,4-glycosidic bonds in linear chains and α-1,6-glycosidic bonds at branching points, together with a small but significant amount of proteins (Wang et al., 2019). Glycogen structure is generally divided into three levels: small γ particles (∼3 nm), spherical β particles (∼20 nm in diameter), and rosette-shaped α particles (up to 300 nm in diameter). α particles are formed by the aggregation of β particles (Wang and Wise, 2019; Liu et al., 2020). Previously, it was thought that only β particles existed in bacteria while α particles were exclusively present in higher organisms, until recently α particles were also observed in *Streptomyces venezuelae* and characterized in *Escherichia coli* (Wang and Wise, 2019; Wang et al., 2019). Some studies reported that the larger glycogen α particles were degraded more slowly than the smaller β particles (Jiang et al., 2016). In addition, chain length distribution patterns of glycogen β particles have also been reported to influence degradation rate (Wang and Wise, 2011). However, formation mechanisms of α particles and determinants of chain length distribution patterns in both prokaryotes and eukaryotes are not completely resolved yet and require further investigation.

Glycogen plays versatile roles in bacteria (Wilson et al., 2010). In some of the prominent pathogens such as *E. coli* (Jones et al., 2008), *Salmonella enteritis* (Bonafonte et al., 2000), *Mycobacterium tuberculosis* (Sambou et al., 2008), and *Vibrio cholerae* (Bourassa and Camilli, 2009), glycogen accumulation has been linked with colonization and/or pathogenicity (Park, 2015). In addition, several studies also support the roles of glycogen in host-pathogen interactions (Gehre et al., 2016). The major function of glycogen in bacteria is energy reserve, which has been confirmed to promote bacterial environmental survival under starvation conditions (Strange, 1968). Glycogen accumulation also facilitates bacterial viability under abiotic stresses such as low temperature, desiccation, and osmotic pressure, etc. (Seibold and Eikmanns, 2007; Dalmasso et al., 2012; Wang et al., 2015). According to the Sit-and-Wait (S&W) hypothesis, prolonged environmental survival could be positively correlated with pathogen evolution toward higher virulence because it increases the opportunity for pathogen to infect susceptible individuals with less dependence on infected hosts, hence less fitness costs (Walther and Ewald, 2004; Wang et al., 2017). A study on *V. cholerae* strongly supported the hypothesis, which showed that accumulated intracellular glycogen facilitated environmental persistence and transmission of *V. cholerae* between aquatic environment and hosts; meanwhile, glycogen-rich *V. cholerae* are more virulent than the glycogen-deficient counterpart (Bourassa and Camilli, 2009). Thus, glycogen as an energy reserve, could also contribute to the evolution of bacterial virulence.

The metabolism of glycogen has been extensively studied in a variety of bacteria. The genome-wide screening of genes affecting glycogen metabolism through 3985 single-gene knockout mutants (the Keio collection) revealed that 65 genes were involved in the glycogen accumulation in *E. coli* (Eydallin et al., 2007b). In addition, screening of genes with enhanced expression in *E. coli* through ASKA library identified 86 genes responsible for glycogen accumulation (Eydallin et al., 2010). Thus, bacterial glycogen metabolism is a sophisticatedly interacted and regulated network that involves multiple genes and pathways (Wilson et al., 2010). Among these genes, five have been considered as the most important in glycogen metabolism, which are *glgC* [glucose-1-phosphate adenylyltransferase (AGPase)], *glgA* [glycogen synthase (GS)], *glgB* [glycogen branching enzyme (GBE)], *glgP* [glycogen phosphorylase (GP)], and *glgX* [glycogen debranching enzyme (GDE)] (Wang and Wise, 2011). The five genes are located in a single operon *glgBXCAP* in *E. coli* with a sub-operon inside *glgC* gene, the encoded enzymes of which form the classical glycogen metabolism pathway (Montero et al., 2010). In particular, AGPase, GS, and GBE are responsible for glycogen biosynthesis while GP and GDE contribute to glycogen degradation.

Previous studies have investigated the influences of single gene mutants in the glycogen metabolism pathway on glycogen accumulation, glycogen structure, environmental persistence, and biofilm formation in different bacterial species, such as *Azospirillum brasilense*, *V. cholera*, and *Corynebacterium glutamicum*, etc. (Jackson et al., 2002b; Seibold and Eikmanns, 2007; Bourassa and Camilli, 2009; Lerner et al., 2009). Single gene knock-out mutants in *E. coli glgBXCAP* operon have also been explored with a focus on the alteration of glycogen accumulation and structure in different studies with non-consistent conditions (Dauvilleìe et al., 2005; Alonso-Casajuìs et al., 2006; Eydallin et al., 2007a; Park et al., 2011; Wang et al., 2015). A recent study comprehensively analyzed the genome-wide phenotypes of growth, cell morphogenesis, and cell cycle events in *E. coli* by using Keio library, which may facilitate our understanding of the influences of glycogen metabolism related genes on *E. coli* physiology (Campos et al., 2018). However, there is currently a lack of comparative study of how *E. coli* genes in *glgBXCAP* operon functions in terms of glucose consumption, glycogen accumulation and structure, biofilm formation, and environmental stress endurance. In this study, we systematically investigated the gene functions of *glgC*, *glgA*, *glgB*, *glgP*, and *glgX* in *E. coli* BW25113 mutants in terms of glycogen metabolism and bacterial physiology with standardized 1 × M9 minimal medium, which were also compared with wide-type strains *E. coli* BL21(DE3) and *E. coli* BW25113. In sum, this study provides a better understanding of glycogen functions in bacterial physiology through phenotypic characterization.

## Materials and Methods

### *E. coli* Strains and Growth Conditions

All the five *E. coli* mutants used in this study, Δ*glgA*, Δ*glgB*, Δ*glgC*, Δ*glgP*, and Δ*glgX*, together with the wide-type parent strain BW25113, were purchased from Horizon Discovery Ltd. (California, United States) as a whole package of the commercial Keio Collection, a systematic, single-gene knockout mutant collection of *E. coli* non-essential genes via λ-Red Recombination System (plasmids pKD4, pKD46, pCP20). Wild-type strain BL21(DE3) was purchased from Tiangen Biotech Co. (Beijing, China). The presence and absence of the five genes (*glgA*, *glgB*, *glgC*, *glgP*, and *glgX*) in the purchased wild-type and mutated *E. coli* strains were confirmed via PCR. For the results of agarose gel electrophoresis, please refer to Supplementary Figure 1. All the primers used for PCR reactions were present in Supplementary Table 1. 1 × M9 minimal medium with 6.78 g/L Na_2_HPO_4_, 3 g/L KH_2_PO_4_, 1 g/L NH_4_Cl, and 0.5 g/L NaCl (Sigma-Aldrich) supplemented with 0.2, 0.4, 0.8, and 1.6% glucose (Sigma-Aldrich) was used for *E. coli* culture, respectively. Liquid and solid Luria-Bertani (LB) media (Tiangen Biotech Co., Beijing, China) were also used for bacterial recovery from −80°C freezer and biofilm formation assay. Phosphate-buffered saline (PBS buffer, Sigma-Aldrich) was used for environmental stress endurance assay. All the strains were cultured at 37°C with shaking rate at 220 rpm when needed, until otherwise specified.

### Growth Curves and Glucose Consumptions

#### Growth Curves

A single colony of BL21(DE3) was picked from LB agar plate and cultured in 5 ml LB broth for 5 h (37°C, 220 rpm). 1 × M9 minimal medium supplemented with 0.2, 0.4, 0.8, and 1.6% glucose was inoculated with the starting culture at 1:20 ratio and cultured at 37°C with shaking rate at 220 rpm, respectively. At selected time points of 2, 4, 6, 8, 10, 12, 16, 18, 20, and 24 h, OD_600_ values were measured and recorded by spectrophotometer (Thermo Fisher Scientific). OD_600_ readings, together with error bars, were drawn by correlating with corresponding time points for growth curves. For each time point, three independent replicates were repeated. As for the growth curves of the seven strains, that is, BL21(DE3), BW25113, Δ*glgC*, Δ*glgA*, Δ*glgB*, Δ*glgP*, and Δ*glgX*, cultured in 1 × M9 minimal medium supplemented with 0.8% glucose, the same procedure described above was followed.

#### Glucose Consumptions

In order to explore the glucose consumption amount in different cultures by different *E. coli* strains, we first measure the glucose content in wide-type BL21(DE3) 1 × M9 culture supplemented with 0.2, 0.4, 0.6, and 0.8% glucose at 0, 4, 6, 10, 12, 14, 16, 18, 20, and 24 h through GOPOD assay kit (Megazyme, Ireland) according to the manufacturer’s instructions. 1 × M9 minimal medium with 0.8% glucose was then used as a standard culture, in which the glucose consumption amount for all the seven strains was measured by following the growth curves. All the experiments were repeated independently for three times.

### Glycogen Quantification, Isolation, and Purification

Glycogen content was assayed for BL21(DE3) along growth curves at 4, 6, 10, 14, 16, 18, 20, and 24 h in 1 × M9 minimal medium supplemented with 0.2, 0.4, 0.8, and 1.6% glucose. As for BW25113 and the five single gene mutated strains, glycogen content and protein amount were assayed at the same time points supplemented with 0.8% glucose only. Three independent replicates were used for each measurement. For glycogen isolation and purification, sucrose gradient density ultracentrifugation (SGDU) was used. All the procedures were followed as previously described (Wang et al., 2015, 2019).

### Glycogen Primary Structure Characterization

Fluorophore-assisted carbohydrate electrophoresis (FACE) was used for characterizing glycogen primary structure (chain length distribution). In particular, 200 unit/mg isoamylase (Megazyme, Ireland) was used to break down all α-1,6-glycosidic branching points in glycogen, which were then labeled with APTS (8-aminopyrene-1,3,6-trisulfonate). The distributions of the number of chains as a function of the degree of polymerization X of that chain after debranching, *N*_de_(X) were measured by standard FACE method. For details, please refer to Wang et al. (2019). The average chain length (ACL) of glycogen was computed by following the previously reported formula (Wang et al., 2015).

### Biofilm Formation Assay

A single bacterial colony was picked up from LB agar to inoculate 5 ml LB broth, which was then cultured at 37°C overnight with 220 rpm shaking rate. 100 μl of the LB broth was inoculated into 10 ml 1 × M9 minimal medium (0.8% glucose) and LB broth, respectively. Each diluted culture was aliquoted to a 96-well microplate (Greiner CELLSTAR^®^ 96 well plates, polystyrene, flat bottom with lid, sterile) with 200 μl/well. Sterile media were added as blank control, correspondingly. The 96-well microplate was cultured statically at 30°C for 20 h. After that, liquid waste was discarded, and planktonic cells were washed out with distilled water for three times. Then, 200 μl 0.1% crystal violet was pipetted into each well, including blank control, for 10 min staining. After that, crystal violet liquid was shaken out of the plate over the waste tray and all plates were washed for three times with distilled water. Finally, all plates were air-dried. 200 μl of 20%/80% ethanol/acetone solution was added to each well for dissolving crystal violet in biofilm for 10 min, 150 μl of which at each well was transferred to a new 96-well plate and OD_590_ value was read and recorded in spectrophotometer.

### Assays for Environmental Stress Resistance

#### Starvation Viability Assay

A single bacterial colony was picked up from LB agar plate for inoculating 5 ml LB medium at 37°C with 220 rpm shaking rate, which was then used to inoculate 100 ml 1 × M9 medium supplemented with 0.8% glucose. The inoculated culture was incubated for 20 h (37°C, 220 rpm), centrifuged at 6000 × *g* for 10 min, and the harvested cells were washed three times with PBS buffer. Cells were then re-suspended in 1 ml PBS buffer, and placed on benchtop at room temperature for 0, 2, 4, 6, 8, 10, and 12 day. The number of viable cells for each time point was calculated via the Miles and Misra method (Miles et al., 2009). All the experiments were independently repeated three times for each strain.

#### Low Temperature Viability Assay

For each studied strain, a single bacterial colony was picked up from LB agar plate for inoculating 5 ml LB medium at 37°C with 220 rpm shaking rate, which was then used to inoculate 100 ml 1 × M9 medium supplemented with 0.8% glucose. After the inoculated culture was incubated for 20 h (37°C, 220 rpm), it was dispended into 1.5 ml Eppendorf tubes with 1 ml/tube and store at 4°C. At day 0, 3, 6, and 9, the suspension was serially and aseptically diluted in a hood by mixing 100 μl diluted culture with 900 μl PBS buffer. For each dilution, Miles and Misra method was used and three replicates were used (Miles et al., 2009). After inoculating, the plate was left in hood to air-dry aseptically, which generally took 20–30 min. When plates were completely dry, plates were incubated at 37°C for 24 h and emerged colonies were counted. The number of viable cells in original culture could then be calculated. As for low temperature viability coupled with starvation viability assay, all the procedures were the same as described above except that after the inoculated culture was incubated for 20 h (37°C, 220 rpm), it was then centrifuged at 6000 × *g* for 10 min, and the harvested cells were washed three times and resuspended in PBS buffer.

#### Desiccation Resistance Assay

Bacterial cell culture was prepared as described in section “Starvation Viability Assay.” After cells were resuspended in 100 ul PBS buffer, 15 μl of concentrated bacterial solution was added to sterilized petri dish for air-dry inside hood. At 0, 1, 3, and 6 h, dried cells were re-suspended in 1.5 ml PBS buffer and continuously diluted from 10^–2^ to 10^–6^. For each dilution, 10 μl of the diluent was taken and dropped onto LB agar plate, which was incubate at 37°C for 24 h. The number of bacterial colonies was counted, and the number of viable cells in original culture was calculated as stated in section “Low Temperature Viability Assay.” The experiments were repeated three times for each strain.

#### Oxidative Stress Resistance Assay

Cells were prepared in the same way as for starvation viability assay. After cultured for 20 h, 2 ml of bacterial solution was mixed with 18 ml LB broth containing 0.8% agarose agar and 1 × M9 minimal medium (0.8% glucose) containing 0.8% agarose agar for agar plate, respectively. 10 μl of 6.6 mol/L H_2_O_2_ was added onto a 6 mm sterilized round filter paper, which was then placed in the center of the agar plate. After culturing the plate statically at 37°C for 24 h, diameter of bacteriostatic ring from three different angles was measured and recorded. For each strain, the experiment was repeated independently three times.

### Statistical Analysis

A two-tailed unequal variance Student *t*-test was calculated for pairwise comparison wherever applicable, unless otherwise instructed. Significant difference is denoted with asterisk(s) when the *P*-value was less than 0.05 (^∗^, *P* < 0.05; ^∗∗^, *P* < 0.01; ^∗∗∗^, *P* < 0.001). The single-step multiple comparison procedure and statistical test, Tukey’s Honestly Significant Difference (HSD) test, was performed for biofilm formation ability and oxidative stress resistance ability among wild-type and mutated strains, which compared the means of every group to the means of every other group simultaneously. Means denoted by a different letter indicated significant differences between groups (*P* < 0.05).

## Results

### Growth Curves

**FIGURE 1:**
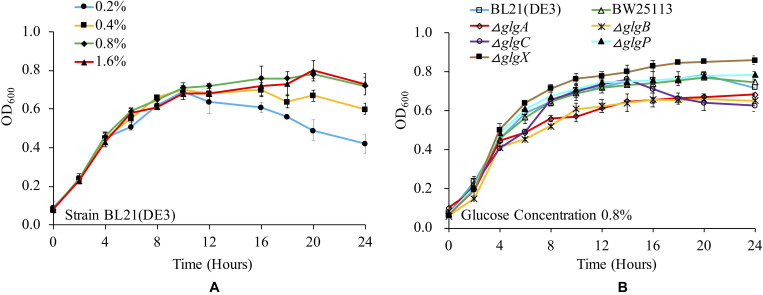
Medium- and strain-dependent *E. coli* growth curves. **(A)** Influences of glucose concentrations in 1 × M9 minimal medium on *E. coli* BL21(DE3) growth. **(B)** Comparison of growth curves of wild-type and mutated *E. coli* strains in 1 × M9 minimal medium supplemented with 0.8% glucose.

Previous studies have already shown that glucose concentration is important in determining bacterial growth and glycogen accumulation (Wang et al., 2018). There are a variety of culture media used for studying glycogen metabolism and accumulation in *E. coli*, which involves the supplementation of complex trace elements and different concentrations of glucose (Wang et al., 2018). In this study, we checked the influence of 1 × M9 minimal medium supplemented with 0.2, 0.4, 0.8, and 1.6% glucose on the growth of *E. coli* BL21(DE3). The results showed that the bacterium had a similar growth curve when cultured in 1 × M9 minimal medium with 0.8 and 1.6% glucose. In contrast, OD_600_ value decreased more rapidly after entering into stationary phase in 1 × M9 minimal medium with 0.2 and 0.4% glucose (Figure 1A). It is also noteworthy that due to the limited nutrients in the minimal medium, the overall OD_600_ values were comparatively lower than LB broth cultures (Wang et al., 2015). Two-tailed unequal variance Student’s *t*-test showed that no significant differences could be detected among all the growth curves through pair-wise comparisons in terms of overall growth trend (*P* > 0.05). However, by comparing OD_600_ values at each time point, statistical significance could be detected from 16 h. In particular, at 24 h, the OD_600_ value of BL21(DE3) in 1 × M9 minimal medium with 0.2% glucose was significantly lower than OD_600_ values of BL21(DE3) growing in other three media (*P* < 0.01) while OD_600_ values of BL21(DE3) in 1 × M9 minimal medium with 0.4% glucose were significantly lower than OD_600_ values of BL21(DE3) growing in 1 × M9 minimal medium with 0.8 and 1.6% glucose (*P* < 0.05). No significant difference was detected between OD_600_ values of BL21(DE3) growing in 1 × M9 minimal medium with 0.8 and 1.6% glucose. Thus, glucose concentration in the culture media has direct impact on *E. coli* growth pattern.

We then cultured wild-type strains BL21(DE3) and BW25113, together with the other five single-gene mutants (Δ*glgC*, Δ*glgA*, Δ*glgB*, Δ*glgP*, and Δ*glgX*) in 1 × M9 minimal medium supplemented with 0.8% glucose, and compared their growth curves (Figure 1B). According to the results, all the strains have similar growth trends. However, two mutants, Δ*glgP* and Δ*glgX*, reached higher OD_600_ values at 24 h than other strains, where Δ*glgX* had the highest OD_600_ value. Student’s *t*-test also showed that there was a significant difference between the two strains at 24 h (*P* < 0.05). As for the two wild-type strains, BL21(DE3) and BW25113, they had similar growing pattern as Δ*glgP* until 20 h. Then, their OD_600_ values dropped evidently. No significant difference was detected for the two wild-type strains in terms of OD_600_ values at 24 h (*P* > 0.05). As for the other three mutants, Δ*glgC*, Δ*glgA*, Δ*glgB*, that were disrupted on glycogen biosynthesis pathway, they showed lowest OD_600_ values at 24 h. In addition, no significant difference was detected among the three strains. Interestingly, Δ*glgC* had similar growing pattern as Δ*glgP* until 14 h, after which its OD_600_ value decreased sharply to the lowest level at 24 h. Student’s *t*-test showed that OD_600_ values of Δ*glgC* mutant at 24 h was significantly lower than BW25113 (*P* < 0.05), Δ*glgP* (*P* < 0.01), and Δ*glgX* (*P* < 0.001). Thus, disruption of genes in glycogen metabolism pathway changed *E. coli* growth abilities. That is, growth of mutants with impaired glycogen degradation pathway was boosted while growth of mutants with compromised glycogen synthesis pathway was hindered.

### Glucose Consumption

**FIGURE 2:**
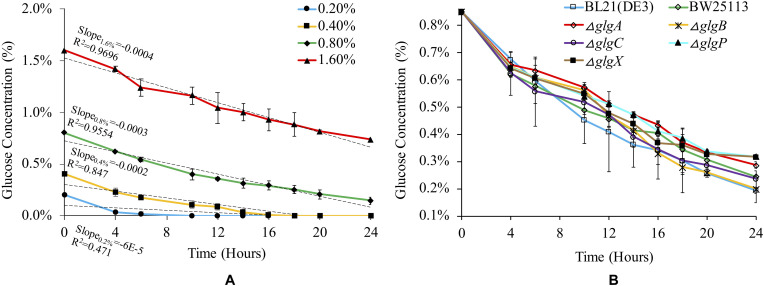
Medium- and strain-dependent glucose consumption rates. **(A)** Glucose consumption rate of *E. coli* BL21(DE3) in 1 × M9 minimal medium supplemented with different percentages of glucose (0.2, 0.4, 0.8, and 1.6%). **(B)** Glucose consumption rate of different *E. coli* strains in 1 × M9 minimal medium supplemented with 0.8% glucose.

Glucose consumption rate for BL21(DE3) was monitored in 1 × M9 minimal medium with different concentrations of glucose (Figure 2A). According to linear regression and statistical analysis, higher glucose concentration leads to slightly but significantly higher rate of glucose consumption among the four groups (*P* < 0.05), except for the comparison between 0.2 and 0.4% glucose (*P* > 0.05). We then checked glucose consumption rates of different strains in 1 × M9 minimal medium with 0.8% glucose (Figure 2B). The results suggested that glucose consumption patterns fluctuated in all the seven strains with an overall decreasing trend. No statistically significant differences were detected among them (*P* > 0.05). However, at 24 h, we noticed that glycogen over-accumulating mutants Δ*glgP* (0.27%) and Δ*glgX* (0.27%) had higher level of glucose left in the liquid culture while glucose left in Δ*glgA* culture (0.24%) is also comparatively high. Thus, disruption of glycogen metabolism pathway has impacts on the rate of glucose consumption in the culture. However, the specific mechanisms for each mutant might be different, which requires further experimental investigations.

### Glycogen Accumulation

**FIGURE 3:**
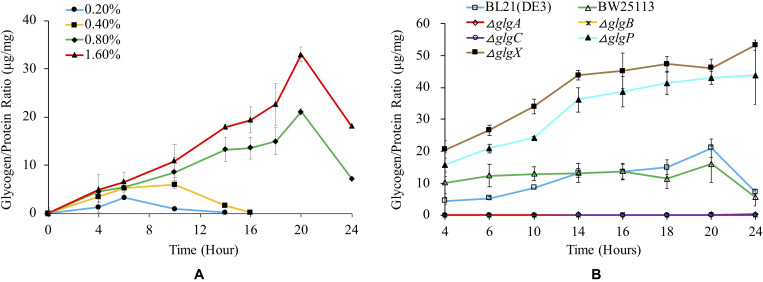
Quantification of glycogen accumulation in *E. coli* strains with different media. **(A)** Glycogen accumulation of *E. coli* BL21(DE3) in 1 × M9 minimal medium supplemented with different percentages of glucose (0.2, 0.4, 0.8, and 1.6%). **(B)** Glycogen accumulation of a variety of *E. coli* strains in 1 × M9 minimal medium supplemented with 0.8% glucose. In order to remove the influence of the biomass change during bacterial growth, glycogen amount (μg) was adjusted by total protein amount (mg). Three independent replicates were performed, and error bars were present.

Glycogen is normally accumulated in bacteria under limited nitrogen source and abundant carbon source (Wang et al., 2019). It is also reported that glycogen accumulation in many bacteria occurs during stationery phase under conditions of limited growth (Bonafonte et al., 2000). In this study, we firstly investigated how glucose concentration influences glycogen accumulation in BL21(DE3) (Figure 3A). The results show that higher glucose concentration in the medium will lead to significantly higher peak amount of accumulated glycogen in the following order: glycogen_1_._6__% glucose, 20 *h*_ > glycogen_0_._8__% glucose, 20 *h*_ > glycogen_0_._4__% glucose, 10 *h*_ > glycogen_0_._2__% glucose, 6 *h*_ (*P* < 0.05). In specificity, as for media supplemented with 0.8 and 1.6% glucose, glycogen accumulation starts from the beginning until 20 h, after which glycogen begins to degrade. In contrast, glycogen accumulation pattern is very different in media supplemented with 0.2 and 0.4% glucose, in which the peak glycogen amount arrives at 6 and 10 h, respectively. No glycogen could be detected at 14 and 16 h due to degradation processes.

We then studied how glycogen accumulation pattern changed in *E. coli* wild-type and single-gene mutants in 1 × M9 minimal medium with 0.8% glucose (Figure 3B). According to the results, three mutated strains (Δ*glgC*, Δ*glgA*, Δ*glgB*) disrupted in glycogen synthesis pathway showed non-detectable glycogen accumulation while two mutated strains (Δ*glgP* and Δ*glgX*) disrupted in glycogen degradation pathway accumulated abnormally higher amount of glycogen than that in wild-type strains BL21(DE3) and BW25113. The general trend of glycogen accumulation level in Δ*glgP* and Δ*glgX* mutants kept increasing during the 24 h culture. There is no statistical significance between the two mutants in terms of overall trend of glycogen accumulation (*P* > 0.05). As for the glycogen accumulation pattern in the two wild-type strains, both of them showed similar pattern, reaching peak value at 20 h and then starting to decline due to degradation. There is also no significant difference identified (*P* > 0.05). However, when comparing wild-type strains, BW25113 and BL21(DE3), and the mutated strains, Δ*glgP* and Δ*glgX*, pair-wisely, the difference is statistically significant (*P* < 0.001).

### Glycogen Primary Structure

**FIGURE 4:**
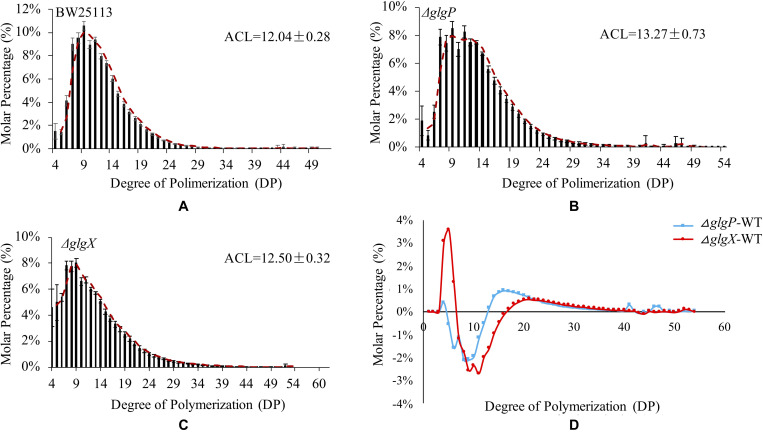
Comparison of glycogen primary structures in *E. coli* wild-type and mutated strains. **(A)** Glycogen chain length distribution patters in *E. coli* BW25113. **(B)** Glycogen chain length distribution patters in *E. coli* BW25113 Δ*glgP*. **(C)** Glycogen chain length distribution patters in *E. coli* BW25113 Δ*glgX*. **(D)** Differential distributions of chain-length molar percentages among wild-type BW25113, Δ*glgP*, and Δ*glgX*.

Previous studies confirmed that glycogen structure was co-ordinately controlled by the *glgBXCAP* operon (Wang and Wise, 2011). Deletion of *glgA* and *glgC* genes had no accumulated glycogen while deletion of *glgB* leads to accumulation of amylose-like linear polysaccharides. Thus, we only compared glycogen primary structure in three strains BW25113, Δ*glgP* and Δ*glgX* (Figure 4). The isoamylase-debranched linear chains showed a typically near bell-shaped distribution pattern for all the three strains. The ACL of glycogen are 12.04 ± 0.28 DP, 13.27 ± 0.73 DP, and 12.5 ± 0.32 DP while all the chain lengths at 9 DP have the highest molar percentages of 10.59, 8.5, and 8% for BW25113, Δ*glgP* and Δ*glgX*, respectively. Differential plot was generated by subtracting the molar percentages of the respective Δ*glgP* and Δ*glgX* oligosaccharide DPs from the corresponding WT molar percentages (Figure 4D). In particular, a significant reduction in the proportion of shorter chains from 5 to 12 DP was observed while the percentage of longer chains with DP equal to or larger than 13 DP were significantly increased in Δ*glgP*. For glycogen chain length distributions in Δ*glgX*, both molar percentage of shorter chains from 4 to 6 DP and that of longer chains equal to or larger than 17 DP were increased while the molar percentage of oligosaccharide chains from 7 to 16 were greatly reduced.

### Biofilm Formation Ability

**FIGURE 5:**
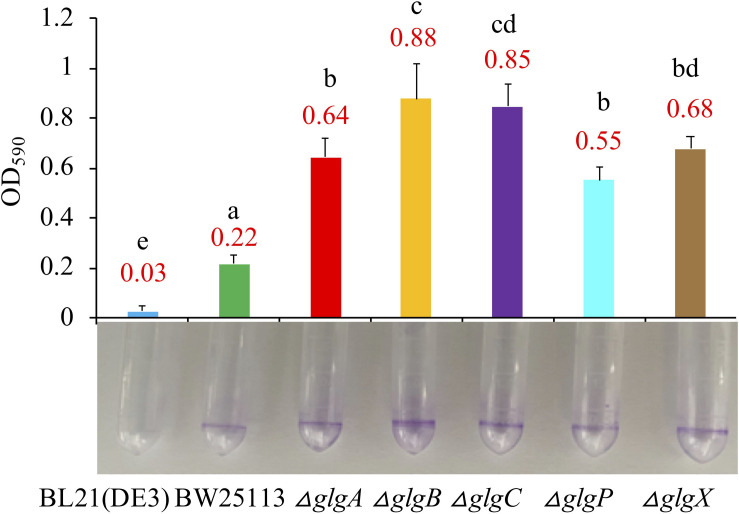
Comparison of biofilm formation abilities of *E. coli* wild-type strains, BL21(DE3) and BW25113, and five mutated strains, Δ*glgA*, Δ*glgB*, Δ*glgC*, Δ*glgP*, and Δ*glgX* in Luria-Bertani broth medium. Biofilm formation abilities in 2 mL plastic round-bottom EP tubes stained with 0.1% crystal violet solution were matched with bar chart that corresponded to mean values and standard errors. Tukey’s HSD test was performed for statistical analysis. Means denoted by a different letter indicated significant differences between groups (*P* < 0.05).

Biofilm formation abilities were compared among the two wild-type strains and five mutants in LB broth medium (Figure 5) and 1 × M9 minimal medium supplemented with 0.8% glucose (Supplementary Figure 2). According to the Tukey’s HSD test, *E. coli* and the mutated strains growing in LB broth formed significantly more biofilms than in M9 minimal medium (*P* < 0.05). When growing in LB broth, BL21(DE3) formed significantly less biofilm than BW25113 (*P* < 0.05). In addition, all mutated strain had significantly higher biofilm formation abilities than that of BW25113 (*P* < 0.05). In particular, Δ*glgB* had the highest biofilm formation potential (OD_590_ = 0.88). When growing in 1 × M9 minimal medium supplemented with 0.8% glucose, biofilm formation abilities of all the *E. coli* strains were significantly reduced to very low level (*P* < 0.05). In sum, 1 × M9 minimal medium (0.8% glucose) did not facilitate the formation of biofilms in *E. coli* while interruption of glycogen metabolism pathway in *E. coli* leads to increased biofilm formation. However, no specific molecular mechanisms are available to interpret such phenotype changes, which we aim to explore via transcriptomics analysis in follow-up studies.

### *E. coli* Viabilities Under Environmental Stresses

**FIGURE 6:**
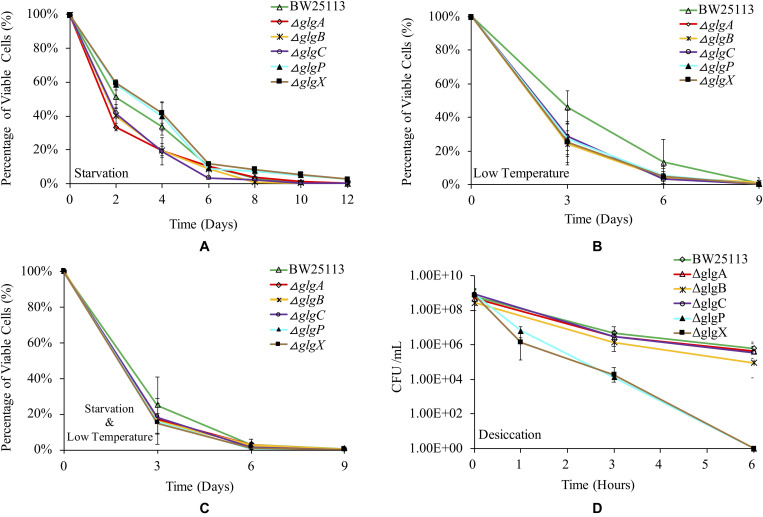
Viabilities of *E. coli* BW25113 wide-type strain and mutated strains under a variety of environmental stress. **(A)** Starvation. **(B)** Low temperature. **(C)** Starvation coupled with low temperature. **(D)** Desiccation.

Glycogen accumulation has been confirmed to facilitate bacterial environmental survival under abiotic stresses (Wang and Wise, 2011). In this study, we systematically studied how the deletions of single genes in the classical *glgBXCAP* operon influence *E. coli* survival under a variety of environmental stresses. Such studies could improve our understanding of glycogen functions further. Parental strain BW25113 and the five single-gene knock-out mutants were re-suspended for starvation in PBS buffer at room temperature for 12 days, during which viable cells in each strain were numerated every 2 days and the results are shown in Figure 6A. It is obvious to see that Δ*glgX* survives the best than all other strains under the starvation conditions for 12 days, which was tightly followed by Δ*glgP* (S_Δ_*_*glgX*_* > S_Δ_*_*glgP*_*). There is no significant difference between Δ*glgX* and Δ*glgP* in terms of the overall trend of starvation survival and when compared at each time point. Viability of the wild-type strain BW25113 is generally lower than Δ*glgX* and Δ*glgP* during the first 4-day starvation, but higher than the other three mutants with glycogen-deficient phenotype (S_Δ*glgX*_ > S_Δ*glgP*_ > S_*WT*_ > S_Δ__*glgCor* Δ*glgBor* Δ*glgA*_). At day 6, viabilities of all the strains clustered together except for Δ*glgC* that had the lowest viability. In addition, wild-type strain survived better than Δ*glgP* with no statistical significance at day 6. Since it is already known that Δ*glgX* and Δ*glgP* store the highest and the second highest amount of glycogen, it is rational to conclude that glycogen accumulation improves bacterial survival under starvation conditions.

As for the survival ability of *E. coli* strains at low temperature, the wild-type BW25113 showed consistently higher viability than all mutated strains until day 9, after which no bacterial viability could be detected for all the strains (Figure 6B). This indicated that disruption of glycogen metabolism pathway led to compromised survival ability of *E. coli* when dealing with cold stress. However, statistical analysis indicated that viability of wild-type BW25113 was only significantly higher than Δ*glgA* and Δ*glgX* at day 3 (*P* < 0.05). No significant difference was detected in terms of the overall trend of cold viability among *E. coli* BW25113 and the five mutants. We then tested the dual effects of starvation and low temperature on the survival rate of *E. coli*. According to the result, wild-type BW25113 had the best viability until day 6 (Figure 6C). Similarly, no statistical significance was found in terms of overall viability under low temperature and starvation environment among *E. coli* strains. Interestingly, the overall survival rate decreased more rapidly when compared with the effects of either starvation or low temperature. At day 9 and beyond, no viable strains could be identified based on the current method.

Desiccation is a strong selective stress on bacterial survival ability during their living in the environment. Due to the rapid death of *E. coli* strains under desiccation condition, we only investigated their viabilities within 6 h (Figure 6D). According to the result, wild-type BW25113 survives the best under desiccation stress. After 6 h, viable cells of wild-type BW25113 dropped from around 10^9^ CFU/ml to 10^6^ CFU/ml. The three glycogen-deficient mutants (Δ*glgC*, Δ*glgA* and Δ*glgB*) decreased their survival abilities more rapidly when compared with the wild-type strain, among which Δ*glgB* performed the worst. As for the mutated strains Δ*glgP* and Δ*glgX*, they died off very quickly, which dropped from around 10^9^ CFU/ml to 10^4^ CFU/ml within only 3 h, and then to 0 CFU/ml within 6 h. The two mutants Δ*glgP* and Δ*glgX* formed an obviously separated cluster from other strains in terms of desiccation viabilities. However, no statistical significance was found among strains in terms of overall survival of desiccation stress.

**FIGURE 7:**
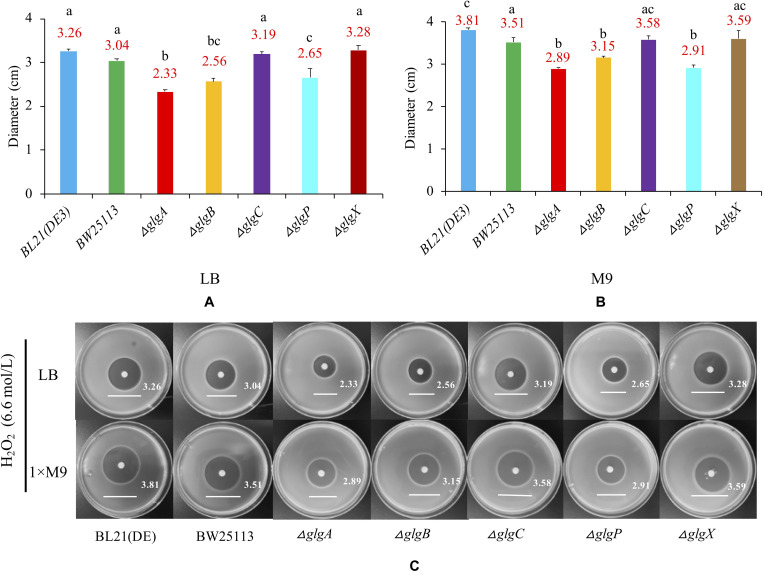
Oxidative stress resistance of *E. coli* strains. **(A)** LB agar plate. **(B)** 1 × M9 minimal medium supplemented with 0.8% glucose agar plate. **(C)** Strain-specific growth inhibition circles. Wild-type strains: BL21(DE3), BW25113. Mutants: Δ*glgC*, Δ*glgA*, Δ*glgB*, Δ*glgP*, and Δ*glgX*. Tukey’s HSD test was performed for statistical analysis. Means denoted by a different letter indicated significant differences between groups (*P* < 0.05).

In order to determine whether the genes in *glgBXCAP* operon could affect the growth of *E. coli* under the oxidative stress condition, bacteria were cultured in LB agar plates and 1 × M9 minimal medium supplemented with 0.8% glucose agar plate. A 6 mm sterilized paper disc contained 10 μl of 6.6 mol/L H_2_O_2_ was then placed in the center of each plate. The diameters of inhibition zones were recorded and compared (Figure 7). According to the statistical analysis, wild-type BW25113 (diameter = 3.51 cm) is slightly but significantly more resistant to H_2_O_2_ than wild-type BL21(DE3) (diameter = 3.81 cm) when cultured in 1 × M9 minimal medium (0.8% glucose) agar plate. As for the five single-gene mutants, oxidative stress resistance was consistently and significantly increased in Δ*glgA*, Δ*glgB*, and Δ*glgP* strains (*P* < 0.05) but insignificantly decreased in Δ*glgC* and Δ*glgX* strains (*P* > 0.05) in both LB and 1 × M9 minimal medium agar plates, when compared with wild-type strain BW25113.

## Discussion

Glycogen accumulation has been linked with bacterial growth, biofilm formation, and environmental survival abilities in a variety of species, some of which are important human pathogens (Seibold and Eikmanns, 2007; Bourassa and Camilli, 2009; Lerner et al., 2009). According to previous studies, many genes were associated with glycogen metabolism in *E. coli* (Eydallin et al., 2007b, 2010). Among these genes, five of them are very important and form a single operon *glgBXCAP* with an alternative suboperonic promoter within *glgC* that directs g*lgAP* expression (Montero et al., 2010). Although these genes have been studied sporadically and scatteringly in different species under different conditions, there is currently no systematic and comparative studies for their physiological functions in *E. coli*. In this study, we cultured *E. coli* wild-type strains, BL21(DE3) and BW25113, and mutated strains, Δ*glgC*, Δ*glgA*, Δ*glgB*, Δ*glgP*, and Δ*glgX*, in LB broth medium and standardized 1 × M9 minimal medium, with a focus on the functions of the five genes in bacterial growth, glycogen accumulation and structure, biofilm formation and environmental survival abilities.

Many studies supported that bacterial glycogen storage and over-accumulation could lead to a better growth or a higher OD_600_ value (Strange, 1968; Dedhia et al., 1994). However, *E. coli* BW25113 Δ*glgX* showed no growth advantage in 1 × M9 medium supplemented with 0.4% glucose, though it accumulated excessive glycogen (Dauvilleìe et al., 2005). The influence of glycogen deficiency or complete depletion on bacterial growth is also disputable. The growth of *C. glutamicum* and its survival in the stationary phase were not altered with complete loss of intracellular glycogen due to inactivation of Δ*glgC* (Seibold et al., 2007). In addition, growth curve of *E. coli* DH5αΔ*glgB* is nearly the same as wild-type strain (Wang et al., 2015). However, *V. cholerae* Δ*glgC* exhibits growth defects during growth under high and low nitrogen conditions (Bourassa and Camilli, 2009). Recently, a genome-wide phenotypic analysis via screening *E. coli* Keio collection reveals that glycogen-excess strains Δ*glgP* (OD_600_ = 0.76) and Δ*glgX* (OD_600_ = 0.76) have higher maximal OD_600_ values than glycogen-deficient strains Δ*glgC* (OD_600_ = 0.69), Δ*glgA* (OD_600_ = 0.43), and Δ*glgB* (OD_600_ = 0.61) (Campos et al., 2018). In this study, we reached similar conclusions: Δ*glgP* and Δ*glgX* had a clearly better growth than the wild-type strain that grew better than other glycogen-deficient mutants (Figure 1). It is worth mentioning that the growth curve of strain Δ*glgC* is similar to wild-type strains initially and then starts to decrease at 14 h. Such a growth defect was previously reported for *V. cholerae* ΔglgC1 and ΔglgC1/Δ*glgC2* mutants when compared with wild-type strain (Bourassa and Camilli, 2009). The possible explanation for this is that growth of *V. cholerae* halts under glycogen−inducing conditions even when glycogen cannot accumulate (Bourassa and Camilli, 2009). However, molecular mechanisms for this phenotype are not clear, which should be explored in future studies. In addition, we also observed that at 24 h, Δ*glgP* and Δ*glgX* had the highest glucose concentration left in the culture, which suggested that glycogen accumulation in Δ*glgP* and Δ*glgX* was due to the disruption of glycogen degradation pathway, rather than higher glucose absorption and utilization rate.

Glycogen accumulation and structure have been widely studied in many bacteria (Wang and Wise, 2011; Wang et al., 2020). In general, bacteria could synthesize glycogen during exponential growth or in stationary phase, but accumulation mainly occurs in excess of carbon source during the stationary phase (Bonafonte et al., 2000; Goh and Klaenhammer, 2014). For wild-type strain *E. coli* BL21(DE3) and BW25113, continuous glycogen accumulation was observed from 4 h until 20 h. Thus, glycogen can be accumulated at exponential phase in *E. coli*, which might be due to low nitrogen concentration in 1 × M9 minimal medium and the high concentration of glucose (0.8%) in the culture. After 20 h culture when glycogen accumulation reaches the peak, its amount starts to decrease sharply, though there was still sufficient glucose left in the medium (Figure 2). Thus, there might be some other regulatory mechanisms that broke the balance of glycogen synthesis and degradation at the stationary phase, which is worthy of further investigation. It was also observed that deletions of Δ*glgC*, Δ*glgA* or Δ*glgB* led to a glycogen loss phenotype, which is consistent with previous reports, while Δ*glgP* or Δ*glgX* knock-out strain caused glycogen over-accumulation with no sign of content reduction.

Glycogen phosphorylase and GDE are responsible for glycogen degradation: GP works by removing glucose units from the non-reducing ends of outer chains in glycogen until four glucose residues left, which are then truncated enzymatically by GDE at α-1,6-glycosidic linkages (Wang and Wise, 2011). Glycogen structures in *E. coli* mutated strains Δ*glgP* and Δ*glgX* have been studied independently (Dauvilleìe et al., 2005; Alonso-Casajuìs et al., 2006). Here we compared the primary structure of glycogen extracted from Δ*glgP* and Δ*glgX* with that in wild-type strain BW25113 after culturing in the same medium for 24 h. The general pattern of structural alterations is consistent with previous conclusions with some variation, which confirms that loss of GP greatly reduces the percentage of short chains while largely increases the percentage of longer chains. As for the deletion of *glgX*, a large portion of very short chains (4–6 DP) were found in the accumulated glycogen particles. However, it is interesting to notice that a certain portion of long chains (17 DP and beyond) also exists, which raises the questions of why these long chains are not degraded by GP and how these chains are organization spatially. Answering these questions would generate a better understanding of glycogen structure and metabolism in bacteria.

Biofilm formation is clinically important because around 80% of microbial infections in the body could be attributed to it (Davies, 2003). There is currently no fixed conclusion in terms of the influences of glucose and glycogen on bacterial biofilm formation (Moreira et al., 2013). A study of *Salmonella enteritidis* revealed that increased glucose concentration in the media facilitated the biosynthesis of intracellular glycogen and biofilm formation (Bonafonte et al., 2000). However, another study showed that biofilm formation was repressed by glucose in several species of *Enterobacteriaceae*, such as *E. coli* (Jackson et al., 2002a). In this study, we identify that BW25113 is a better biofilm former than BL21(DE3) in LB broth. In addition, glycogen metabolism disruption significantly improves biofilm formation in the same medium. In contrast, biofilm formation in 1 × M9 minimal medium supplemented with 0.8% glucose for all strains is strongly and significantly repressed. Thus, biofilm formation is species-dependent and also greatly influenced by environmental factors (Jackson et al., 2002a).

Glycogen accumulation facilitates bacterial environmental survival under a variety of harsh conditions (Klotz and Forchhammer, 2017). In addition, glycogen with short ACL has also been linked with bacterial environmental durability due to its slow degradation rate (Wang and Wise, 2011). In this study, we investigated the influences of glycogen on the survival abilities of *E. coli* BW25113 and its single-gene mutants under starvation, low temperature, desiccation, and oxidative stress. As an important energy reserve, bacterial glycogen was able to provide both short-term benefits in changing environments (Sekar et al., 2020) and long-term persistence in the environment (Bourassa and Camilli, 2009). Our study further confirmed that glycogen over-accumulating strains survived much better than wild-type and other mutated strains under starvation conditions, even though glycogen utilization was disrupted. Such an advantage also suggested that there might be other unknown enzyme(s) that can release glucose and/or maltodextrins from intracellular glycogen (Strydom et al., 2017).

Survival of temperature fluctuation is important for pathogens during prolonged transfer between hosts (Chakravortty et al., 2012). Trehalose is known to be essential for viability of *E. coli* at low temperatures (Kandror et al., 2002). Meanwhile, metabolism of trehalose and glycogen are interconnected through *treS*–*pep2*–*glgE-glgB* pathway (Wang and Wise, 2019). Previous studies have already found that both glycogen and trehalose are highly accumulated as a response to cold stimulation in *Propionibacterium freudenreichii* (Dalmasso et al., 2012). In this study, low temperature (4°C) was imposed on *E. coli* wild-type strain BW25113 and five mutated strains for nine consecutive days, the result of which showed that wild-type strain survived the best while other mutants had a similarly compromised survival. When coupling starvation with low temperature, the general trend was similar as the stress of low temperature alone except that all *E. coli* strains died off much faster. Thus, disruption of glycogen metabolism pathway compromised *E. coli* survival in cold environment, no matter whether glycogen was over- or less accumulated, which suggested that a complete *glgBXCAP* operon was necessary for *E. coli* to combat cold stress.

**FIGURE 8:**
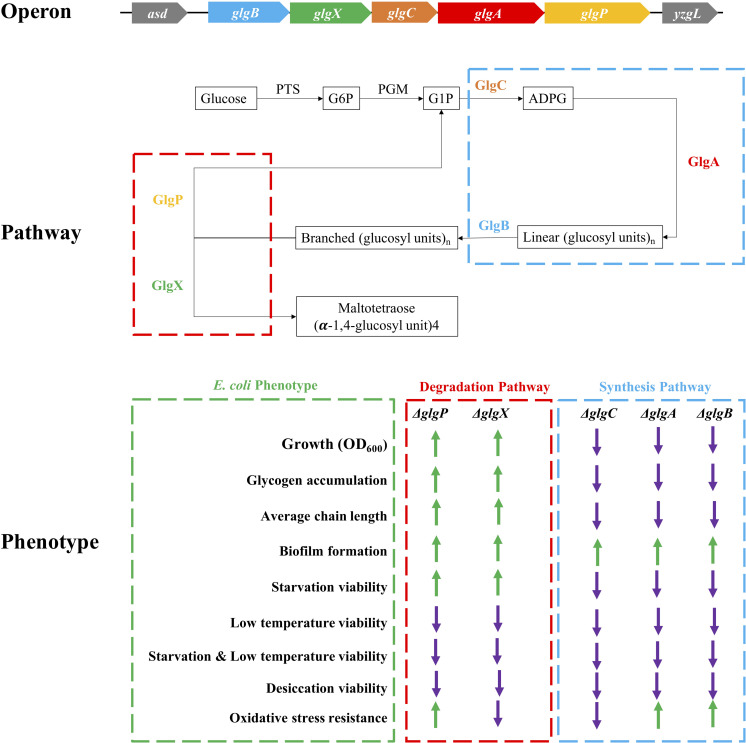
Schematic illustration of *glgBXCAP* operon and the corresponding protein functions in *E. coli* growth, glycogen accumulation and structure, biofilm formation, and environmental stress endurance. ↑: increment when compared to wild-type BW25113 (green arrow); ↓: decrement when compared to wild-type BW25113 (violet arrow).

Desiccation is an environmental and food industry stress that bacteria commonly face, which significantly affects bacterial viability more than other stresses (Esbelin et al., 2018). *glgP* deletion in *A. brasilense* showed significantly higher survival rate than wild-type in 1-h desiccation (Lerner et al., 2009). However, *glgB* deletion in *E. coli* DH5α greatly reduced its survival rate when desiccated for 6 h (Wang et al., 2015). Through systematic comparison of *E. coli* BW25113 and its five single-gene mutants in *glgBXCAP* operon, we found out that wild-type survives the best under the stress while glycogen over-accumulating strains Δ*glgP* and Δ*glgX* were more sensitive to desiccation than the other three mutants. However, there is currently no clear molecular mechanism to explain this difference. As for the oxidative stress, recent studies showed that glucose released from glycogen could be used for NADPH/glutathione reduction, which protects nematodes and human hepatocytes from oxidative stress; however, the accumulation of glycogen itself paradoxically shortens the lifespan of *Caenorhabditis elegans* (Gusarov et al., 2017). In bacteria, oxidative stress resistance normally include two distinct responses, peroxide stimulon and superoxide stimulon, which involves around 44 genes with no direct relationship with glycogen metabolism (Farr and Kogoma, 1991). In this study, there is no consistent conclusion about oxidative resistance in terms of glycogen accumulation and deficiency. Meanwhile, Δ*glgA* unexpectedly showed the highest resistance toward oxidative stress, which requires further experimental investigation. For a complete summary of all these phenotypic changes influenced by the disruption of glycogen metabolism pathway, please refer to Figure 8.

## Conclusion

Taken together, this study systematically and comparatively investigates how single gene deletion in the important and classical *glgBXCAP* operon influences the physiological activities of *E. coli*. Differences have been identified in terms of growth rate, glycogen accumulation and structure, biofilm formation, and environmental stress endurance, which confirms the importance of glycogen metabolism in bacteria. However, molecular basis for some of these phenotypic differences are still missing. Further investigation into the molecular mechanisms are needed, which could provide a better understanding of glycogen functions in bacteria.

## Data Availability Statement

All datasets presented in this study are included in the article/Supplementary Material.

## Author Contributions

LW conceived and designed the experiments. LW, DT, and XZ contributed to project administration. LW, MW, QL, and XK carried out the experimental work. LW, DT, MW, QL, MG, YD, XZ, and XX wrote and revised the manuscript. LW, DT, XZ, ZZ, and HY provided platform, resources, and student supervision. All authors read and approved the final manuscript.

## Conflict of Interest

The authors declare that the research was conducted in the absence of any commercial or financial relationships that could be construed as a potential conflict of interest.

## References

[B1] Alonso-Casajuìs N. Dauvilleìe D. Viale A. M. Munþoz F. J. Baroja-Fernaìndez E. Moraìn-Zorzano M. A. T. (2006). Glycogen phosphorylase, the product of the glgp gene, catalyzes glycogen breakdown by removing glucose units from the nonreducing ends in *Escherichia coli*. *J. Bacteriol.* 188 5266–5272. 10.1128/jb.01566-05 16816199 PMC1539952

[B2] Bonafonte M. A. Solano C. Sesma B. A. Alvarez M. Montuenga L. García-Ros D. (2000). The relationship between glycogen synthesis, biofilm formation and virulence in *Salmonella* enteritidis. *FEMS Microbiol. Lett.* 191 31–36. 10.1111/j.1574-6968.2000.tb09315.x 11004396

[B3] Bourassa L. Camilli A. (2009). Glycogen contributes to the environmental persistence and transmission of *Vibrio cholerae*. *Mol. Microbiol.* 72 124–138. 10.1111/j.1365-2958.2009.06629.x 19226328 PMC2704980

[B4] Campos M. Govers S. K. Irnov I. Dobihal G. S. Cornet F. Jacobs-Wagner C. (2018). Genomewide phenotypic analysis of growth, cell morphogenesis, and cell cycle events in *Escherichia coli*. *Mol. Syst. Biol.* 14:e7573. 10.15252/msb.20177573 29941428 PMC6018989

[B5] Chakravortty D. Onyango L. A. Dunstan R. H. Gottfries J. von Eiff C. Roberts T. K. (2012). Effect of low temperature on growth and ultra-structure of *Staphylococcus* spp. *PLoS One* 7:e0029031. 10.1371/journal.pone.0029031 22291884 PMC3265459

[B6] Dalmasso M. Aubert J. Even S. Falentin H. Maillard M.-B. Parayre S. (2012). Accumulation of intracellular glycogen and trehalose by propionibacterium freudenreichii under conditions mimicking cheese ripening in the cold. *Appl. Environ. Microbiol.* 78 6357–6364. 10.1128/aem.00561-12 22729537 PMC3416590

[B7] Dauvilleìe D. Kinderf I. S. Li Z. Kosar-Hashemi B. Samuel M. S. Rampling L. (2005). Role of the *Escherichia coli* glgX Gene in glycogen metabolism. *J. Bacteriol.* 187 1465–1473. 10.1128/jb.187.4.1465-1473.2005 15687211 PMC545640

[B8] Davies D. (2003). Understanding biofilm resistance to antibacterial agents. *Nat. Rev. Drug Discov.* 2 114–122. 10.1038/nrd1008 12563302

[B9] Dedhia N. N. Hottiger T. Bailey J. E. (1994). Overproduction of glycogen in *Escherichia coli* blocked in the acetate pathway improves cell growth. *Biotechnol. Bioeng.* 44 132–139. 10.1002/bit.260440119 18618456

[B10] Esbelin J. Santos T. Hébraud M. (2018). Desiccation: an environmental and food industry stress that bacteria commonly face. *Food Microbiol.* 69 82–88. 10.1016/j.fm.2017.07.017 28941912

[B11] Eydallin G. Montero M. Almagro G. Sesma M. T. Viale A. M. Munoz F. J. (2010). Genome-wide screening of genes whose enhanced expression affects glycogen accumulation in *Escherichia coli*. *DNA Res.* 17 61–71. 10.1093/dnares/dsp028 20118147 PMC2853380

[B12] Eydallin G. Morán-Zorzano M. T. Muñoz F. J. Baroja-Fernández E. Montero M. Alonso-Casajús N. (2007a). An *Escherichia coli* mutant producing a truncated inactive form of GlgC synthesizes glycogen: further evidences for the occurrence of various important sources of ADPglucose in enterobacteria. *FEBS Lett.* 581 4417–4422. 10.1016/j.febslet.2007.08.016 17719034

[B13] Eydallin G. Viale A. M. Morán-Zorzano M. T. Muñoz F. J. Montero M. Baroja-Fernández E. (2007b). Genome-wide screening of genes affecting glycogen metabolism in*Escherichia coli*K-12. *FEBS Lett.* 581 2947–2953. 10.1016/j.febslet.2007.05.044 17543954

[B14] Farr S. B. Kogoma T. (1991). Oxidative stress responses in *Escherichia coli* and *Salmonella typhimurium*. *Microbiol. Rev.* 55 561–585. 10.1128/mmbr.55.4.561-585.1991 1779927 PMC372838

[B15] Gehre L. Gorgette O. Perrinet S. Prevost M.-C. Ducatez M. Giebel A. M. (2016). Sequestration of host metabolism by an intracellular pathogen. *eLife* 5:e12552. 10.7554/eLife.12552 26981769 PMC4829429

[B16] Goh Y. J. Klaenhammer T. R. (2014). Insights into glycogen metabolism in *Lactobacillus acidophilus*: impact on carbohydrate metabolism, stress tolerance and gut retention. *Microb. Fact.* 13:94. 10.1186/s12934-014-0094-3 25410006 PMC4243779

[B17] Gusarov I. Pani B. Gautier L. Smolentseva O. Eremina S. Shamovsky I. (2017). Glycogen controls *Caenorhabditis elegans* lifespan and resistance to oxidative stress. *Nat. Commun.* 8:15868. 10.1038/ncomms15868 28627510 PMC5481799

[B18] Jackson D. W. Simecka J. W. Romeo T. (2002a). Catabolite repression of *Escherichia coli* biofilm formation. *J. Bacteriol.* 184 3406–3410. 10.1128/jb.184.12.3406-3410.2002 12029060 PMC135108

[B19] Jackson D. W. Suzuki K. Oakford L. Simecka J. W. Hart M. E. Romeo T. (2002b). Biofilm formation and dispersal under the influence of the global regulator CsrA of *Escherichia coli*. *J. Bacteriol.* 184 290–301. 10.1128/jb.184.1.290-301.2002 11741870 PMC134780

[B20] Jiang X. Zhang P. Li S. Tan X. Hu Z. Deng B. (2016). Molecular-size dependence of glycogen enzymatic degradation and its importance for diabetes. *Eur. Polym. J.* 82 175–180. 10.1016/j.eurpolymj.2016.07.017

[B21] Jones S. A. Jorgensen M. Chowdhury F. Z. Rodgers R. Hartline J. Leatham M. P. (2008). Glycogen and maltose utilization by *Escherichia coli* O157:H7 in the mouse intestine. *Infect. Immun.* 76 2531–2540. 10.1128/iai.00096-08 18347038 PMC2423072

[B22] Kandror O. DeLeon A. Goldberg A. L. (2002). Trehalose synthesis is induced upon exposure of *Escherichia coli* to cold and is essential for viability at low temperatures. *Proc. Natl. Acad. Sci. U.S.A.* 99 9727–9732. 10.1073/pnas.142314099 12105274 PMC124994

[B23] Klotz A. Forchhammer K. (2017). Glycogen, a major player for bacterial survival and awakening from dormancy. *Future Microb.* 12 101–104. 10.2217/fmb-2016-0218 28106464

[B24] Lerner A. Castro-Sowinski S. Lerner H. Okon Y. Burdman S. (2009). Glycogen phosphorylase is involved in stress endurance and biofilm formation in *Azospirillum brasilense* Sp7. *FEMS Microbiol. Lett.* 300 75–82. 10.1111/j.1574-6968.2009.01773.x 19765087

[B25] Liu Q. Zhu Z. Wang M. Wang Y. Zhang P. Wang H. (2020). Characterization of glycogen molecular structure in the worm *Caenorhabditis elegans*. *Carbohydr. Polym.* 237:116181. 10.1016/j.carbpol.2020.116181 32241425

[B26] Miles A. A. Misra S. S. Irwin J. O. (2009). The estimation of the bactericidal power of the blood. *Epidemiol. Infect.* 38 732–749. 10.1017/s002217240001158x 20475467 PMC2199673

[B27] Montero M. Almagro G. Eydallin G. Viale Alejandro M. Muñoz, Francisco J. (2010). *Escherichia coli* glycogen genes are organized in a single glgBXCAP transcriptional unit possessing an alternative suboperonic promoter within glgC that directs glgAP expression. *Biochem. J.* 433 107–117. 10.1042/bj20101186 21029047

[B28] Moreira J. M. R. Gomes L. C. Araújo J. D. P. Miranda J. M. Simões M. Melo L. F. (2013). The effect of glucose concentration and shaking conditions on *Escherichia coli* biofilm formation in microtiter plates. *Chem. Eng. Sci.* 94 192–199. 10.1016/j.ces.2013.02.045

[B29] Park J. T. Shim J. H. Tran P. L. Hong I. H. Yong H. U. Oktavina E. F. (2011). Role of maltose enzymes in glycogen synthesis by *Escherichia coli*. *J. Bacteriol.* 193 2517–2526. 10.1128/jb.01238-10 21421758 PMC3133173

[B30] Park K.-H. (2015). Roles of enzymes in glycogen metabolism and degradation in *Escherichia coli*. *J. Appl. Glycosci.* 62 37–45. 10.5458/jag.jag.JAG-2015_005

[B31] Sambou T. Dinadayala P. Stadthagen G. Barilone N. Bordat Y. Constant P. (2008). Capsular glucan and intracellular glycogen of *Mycobacterium tuberculosis*: biosynthesis and impact on the persistence in mice. *Mol. Microbiol.* 70 762–774. 10.1111/j.1365-2958.2008.06445.x 18808383 PMC2581643

[B32] Seibold G. Dempf S. Schreiner J. Eikmanns B. J. (2007). Glycogen formation in *Corynebacterium glutamicum* and role of ADP-glucose pyrophosphorylase. *Microbiology* 153 1275–1285. 10.1099/mic.0.2006/003368-0 17379737

[B33] Seibold G. M. Eikmanns B. J. (2007). The glgX gene product of *Corynebacterium glutamicum* is required for glycogen degradation and for fast adaptation to hyperosmotic stress. *Microbiology* 153 2212–2220. 10.1099/mic.0.2006/005181-0 17600065

[B34] Sekar K. Linker S. M. Nguyen J. Grünhagen A. Stocker R. Sauer U. (2020). Bacterial glycogen provides short-term benefits in changing environments. *Appl. Environ. Microbiol.* 86:e00049-20. 10.1128/aem.00049-20 32111592 PMC7170487

[B35] Strange R. E. (1968). Bacterial “glycogen” and survival. *Nature* 220 606–607. 10.1038/220606a0 4879742

[B36] Strydom L. Jewell J. Meier M. A. George G. M. Pfister B. Zeeman S. (2017). Analysis of genes involved in glycogen degradation in *Escherichia coli*. *FEMS Microbiol. Lett.* 364:fnx016. 10.1093/femsle/fnx016 28119371

[B37] Walther B. A. Ewald P. W. (2004). Pathogen survival in the external environment and the evolution of virulence. *Biol. Rev.* 79 849–869. 10.1017/s1464793104006475 15682873 PMC7161823

[B38] Wang L. Liu Q. Du Y. Tang D. Wise M. (2018). Optimized M9 minimal salts medium for enhanced growth rate and glycogen accumulation of *Escherichia coli* DH5α. *Microbiol. Biotechnol. Lett.* 46 194–200. 10.4014/mbl.1804.04010

[B39] Wang L. Liu Q. Tan X. Wang Z. Wang M. Wise M. J. (2019). Molecular structure of glycogen in *Escherichia coli*. *Biomacromolecules* 20 2821–2829. 10.1021/acs.biomac.9b00586 31244022

[B40] Wang L. Liu Z. Dai S. Yan J. Wise M. J. (2017). The sit-and-wait hypothesis in bacterial pathogens: a theoretical study of durability and virulence. *Front. Microbiol.* 8:2167. 10.3389/fmicb.2017.02167 29209284 PMC5701638

[B41] Wang L. Regina A. Butardo V. M. Kosar-Hashemi B. Larroque O. Kahler C. M. (2015). Influence of in situ progressive N-terminal is still controversial truncation of glycogen branching enzyme in *Escherichia coli* DH5α on glycogen structure, accumulation, and bacterial viability. *BMC Microbiol.* 15:96. 10.1186/s12866-015-0421-9 25947105 PMC4433092

[B42] Wang L. Wang M. Wise M. J. Liu Q. Yang T. Zhu Z. (2020). Recent progress in the structure of glycogen serving as a durable energy reserve in bacteria. *World J. Microbiol. Biotechnol.* 36:14. 10.1007/s11274-019-2795-6 31897771

[B43] Wang L. Wise M. J. (2011). Glycogen with short average chain length enhances bacterial durability. *Naturwissenschaften* 98 719–729. 10.1007/s00114-011-0832-x 21808975

[B44] Wang L. Wise M. J. (2019). An updated view on bacterial glycogen structure. *Microbiol. Austr*. 40 195–199. 10.1071/ma19056

[B45] Wilson W. A. Roach P. J. Montero M. Baroja-Fernández E. Muñoz F. J. Eydallin G. (2010). Regulation of glycogen metabolism in yeast and bacteria. *FEMS Microbiol. Rev.* 34 952–985. 10.1111/j.1574-6976.2010.00220.x 20412306 PMC2927715

