# Supplementary material for: Glycogen Metabolism Impairment via Single Gene Mutation in the glgBXCAP Operon Alters the Survival Rate of Escherichia coli Under Various Environmental Stresses
Source: Front Microbiol. 2020 Sep 25;11:588099. doi: 10.3389/fmicb.2020.588099 (PMC7546213; doi:10.3389/fmicb.2020.588099)
Supplement: Supplementary file 1 [file Image_1.pdf]

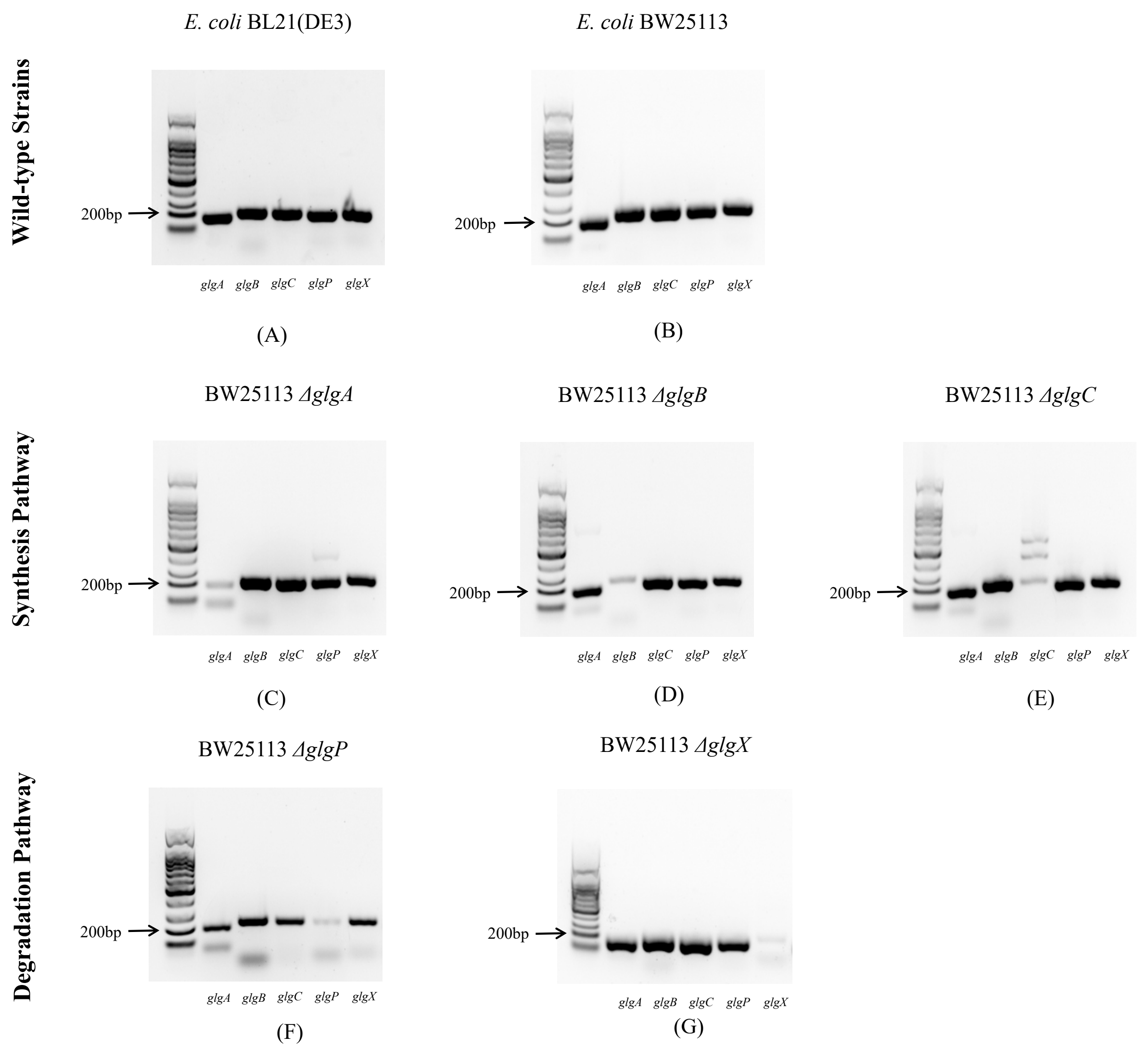

Supplementary Figure 1 PCR verification of two *E. coli* wild-type strains, BL21(DE3) and BW25113, and five commercial *E. coli* BW25113 single-gene knockout mutants (*ΔglgA*, *ΔglgB*, *ΔglgC*, *ΔglgP*, *ΔglgX*) in KEIO collection that was purchased from Horizon Discovery Ltd. https://horizondiscovery.com/. Results confirm that glycogen metabolism relevant genes (*glgA*, *glgB*, *glgC*, *glgP*, *glgX*) are complete in wild-type strains. For each single-gene mutant, the corresponding gene is missing. (A) *E. coli* BL21(DE3) (B) *E. coli* BW25113 (C) *E. coli* BW25113 *ΔglgA* (D) *E. coli* BW25113 *ΔglgB* (E) *E. coli* BW25113 *ΔglgC* (F) *E. coli* BW25113 *ΔglgP* (G) *E. coli* BW25113 *ΔglgX*.
