# Supplementary material for: Glycogen Metabolism Impairment via Single Gene Mutation in the glgBXCAP Operon Alters the Survival Rate of Escherichia coli Under Various Environmental Stresses
Source: Front Microbiol. 2020 Sep 25;11:588099. doi: 10.3389/fmicb.2020.588099 (PMC7546213; doi:10.3389/fmicb.2020.588099)
Supplement: Supplementary file 2 [file Image_2.pdf]

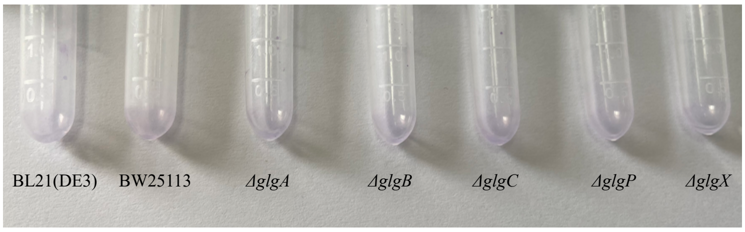

(A)

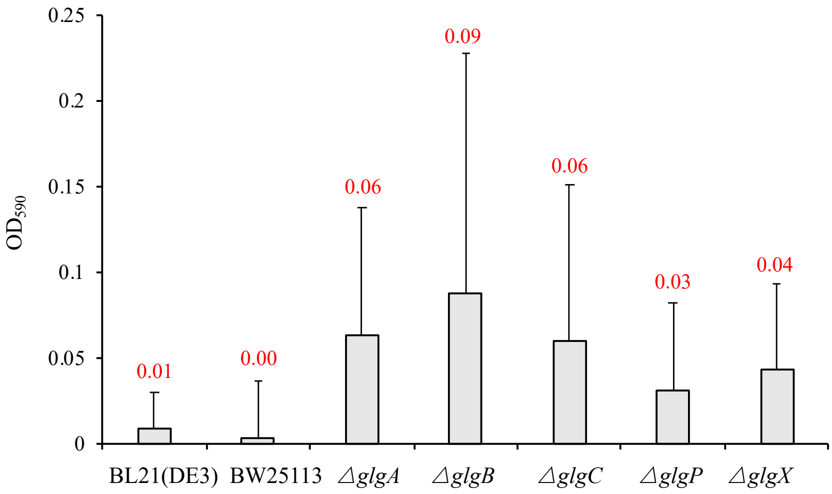

(B)

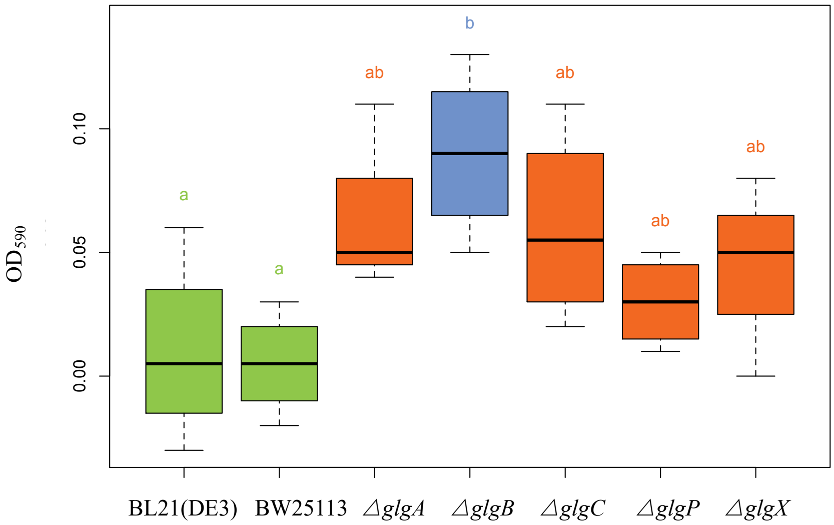

(C)

Supplementary Figure 2 Comparison of biofilm formation abilities of *E. coli* wild-type and mutated strains in 1×M9 minimal medium supplemented with 0.8% glucose. (A) Biofilm formation abilities in 2 mL plastic EP tubes stained with 0.1% crystal violet solution. (B) Crystal violet concentration measured by absorbance at 590 nm after biofilm matrix stain was solubilized in 20%/80% ethanol/acetone solution. (C). Boxplot of Tukey's honest significance test. Groups labelled with the same letter have no statistically significant difference. Groups labelled with different letters indicate statistically significant difference ($p<0.05$). *ab* means no statistically significant difference from *a* or *b*.
