# Supplementary material for: Glycogen Metabolism Impairment via Single Gene Mutation in the glgBXCAP Operon Alters the Survival Rate of Escherichia coli Under Various Environmental Stresses
Source: Front Microbiol. 2020 Sep 25;11:588099. doi: 10.3389/fmicb.2020.588099 (PMC7546213; doi:10.3389/fmicb.2020.588099)
Supplement: Supplementary file 3 [file Table_1.docx]

**Supplementary Table 1** Oligonucleotide primer pairs used in PCR. All the primers were designed by using Primer3Plus platform <http://www.primer3plus.com/cgi-bin/dev/primer3plus.cgi>

| Name | Forward primer (5’-3’) | Reverse primer (5’-3’) |
| --- | --- | --- |
| *glgA* | TGGGCTTGCTGATACGGTTT | CACAAACCGCCACAGTGAAG |
| *glgB* | AGGTTGTACAGACTGAAGAGCG | TGCTCGTTAATGGGCAGTAGTT |
| *glgC* | CAAAACCTCGACATTATCCGCC | GTTCTCATCAACCGCCATAACG |
| *glgP* | TTGCCGACTTTGCGAAAATCTT | AATCACAGTGTTGTTGCAGCTC |
| *glgX* | GCGTTATTGGGTAGAAACCTGC | TTTCCCACCTGATAACCACCAG |
